# 1,3,5-Tris(4-meth­oxy­phen­yl)-1,3,5-triazinane-2,4,6-trione

**DOI:** 10.1107/S160053681303482X

**Published:** 2014-01-08

**Authors:** Li Fang, Feifei Li, Xuemei Luo

**Affiliations:** a The School of Chemistry and Chemical Engineering, Shanxi University, Taiyuan 030006, People’s Republic of China

## Abstract

The complete mol­ecule of the title compound, C_24_H_21_N_3_O_6_, is generated by the application of threefold rotation symmetry about an axis perpendicular to the central ring. The mol­ecule exhibits a propeller-like shape. The dihedral angle between each benzene ring and the heterocyclic ring is 74.0 (1)°. The mol­ecules pack with no specific inter­molecular inter­actions between them. The *SQUEEZE* procedure in *PLATON* [Spek (2009 ▶). *Acta Cryst.* D**65**, 148–155] was used to model disordered solvent mol­ecules, presumed to be acetone; the calculated unit-cell data do not take into account the presence of these.

## Related literature   

For general background to trimerization of aromatic iso­cyanates, see: Raders & Verkade (2010 ▶); Duong *et al.* (2004 ▶); Tang *et al.* (1994 ▶); Zhitinkina *et al.* (1985 ▶); Nawata *et al.* (1975 ▶); Nicholas & Gmitter (1965 ▶).
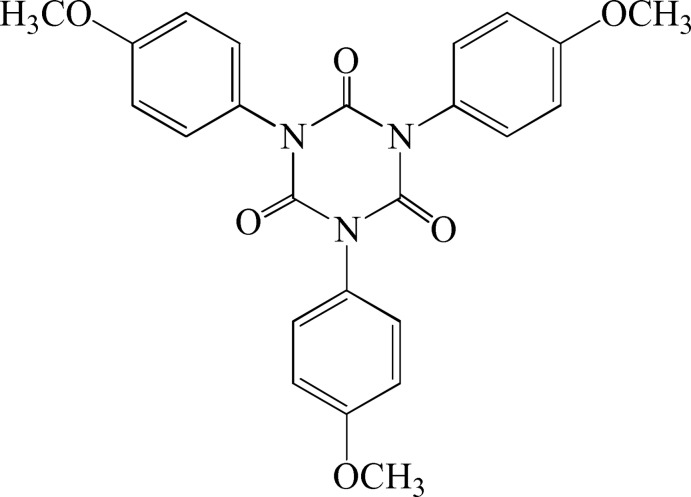


## Experimental   

### 

#### Crystal data  

C_24_H_21_N_3_O_6_
*M* *_r_* = 447.44
Trigonal, 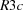

*a* = 13.2008 (14) Å
*c* = 26.695 (3) Å
*V* = 4028.7 (8) Å^3^
*Z* = 6
Mo *K*α radiation
μ = 0.08 mm^−1^
*T* = 200 K
0.51 × 0.49 × 0.04 mm

#### Data collection  

Bruker SMART CCD area-detector diffractometer
Absorption correction: multi-scan (*SADABS*; Sheldrick, 1996 ▶) *T*_min_ = 0.960, *T*_max_ = 0.997
6995 measured reflections
1577 independent reflections
1142 reflections with *I* > 2σ(*I*)
*R*_int_ = 0.031

#### Refinement  

*R*[*F* ^2^ > 2σ(*F* ^2^)] = 0.044
*wR*(*F* ^2^) = 0.122
*S* = 1.05
1577 reflections
101 parameters
1 restraint
H-atom parameters constrained
Δρ_max_ = 0.16 e Å^−3^
Δρ_min_ = −0.12 e Å^−3^

### 

Data collection: *SMART* (Bruker, 2007 ▶); cell refinement: *SAINT* (Bruker, 2007 ▶); data reduction: *SAINT*; program(s) used to solve structure: *SHELXS97* (Sheldrick, 2008 ▶); program(s) used to refine structure: *SHELXL97* (Sheldrick, 2008 ▶); molecular graphics: *SHELXTL* (Sheldrick, 2008 ▶); software used to prepare material for publication: *SHELXTL* and *PLATON* (Spek, 2009 ▶).

## Supplementary Material

Crystal structure: contains datablock(s) I, New_Global_Publ_Block. DOI: 10.1107/S160053681303482X/tk5284sup1.cif

Structure factors: contains datablock(s) I. DOI: 10.1107/S160053681303482X/tk5284Isup2.hkl

Supporting information file. DOI: 10.1107/S160053681303482X/tk5284Isup3.cml

CCDC reference:

Additional supporting information:  crystallographic information; 3D view; checkCIF report

## Figures and Tables

**Fig. 1.**
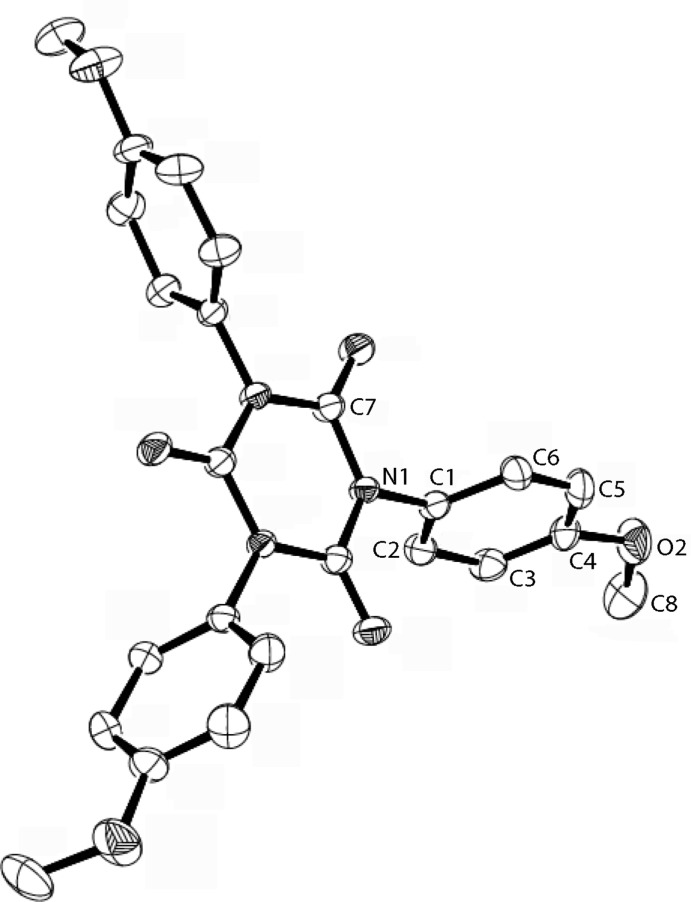
Molecular structure of I showing 30% probability displacement ellipsoids. The hydrogen atoms are omitted for clarity.

## supplementary crystallographic information

### 2.1. Synthesis and crystallization

To a stirred solution of lithium di­benzyl­amide (0.04 g) in di­ethyl ether was added 4-meth­oxy­phenyl iso­thio­cyanate (29.8 g). After stirring for 2 min, the mixture afforded a white precipitate. The resulting precipitate was collected by suction filtration and recrystallized from acetone to obtain white crystals of 1,3,5-tris-(4-meth­oxy­phenyl)-1,3,5-triazinane-2,4,6-trione (yield: 90%), m.p. 530–531 K.

### 2.2. Refinement

The H atoms were placed in their idealised positions with C—H = 0.95–0.98 Å, and refined as riding with *U*_iso_(H) = 1.2–1.5*U*_eq_. The structure contains solvent accessible voids of 153 A^3^. The *SQUEEZE* procedure in *PLATON* (Spek, 2009) was used to model the disordered solvent molecules, presumed to be 4–6 acetone molecules per unit cell.

### 3. Results and discussion

Trimerization of aromatic iso­cyanates, manufactured by cyclo­trimerizing corresponding iso­cyanates, has been known to enhance the properties of polyurethanes or coating materials (Raders & Verkade, 2010; Duong *et al.*, 2004; Tang *et al.*, 1994; Zhitinkina *et al.*, 1985; Nawata *et al.*, 1975; Nicholas & Gmitter, 1965). Polymers, such as polyurethanes incorporated with isocyanurates, have enhanced thermal resistance, flame retardation, chemical resistance and film-forming characteristics. Isocyanurates are also used in the synthesis of co-polymer resins to improve their water-resistance, transparency and impact resistance. During research on the properties of lithium di­benzyl­amide, a simple and efficient catalyst for iso­cyanate cyclo­trimerization to isocyanurate, we obtained crystals of 1,3,5-tris-(4-meth­oxy­phenyl)-1,3,5-triazinane-2,4,6-trione.

In the title compound, C_24_H_21_N_3_O_6_, the six-membered heterocyclic ring lies on a threefold rotation axis and adopts a planar conformation. The molecule exhibits a propeller-like shape. The dihedral angle between each benzene ring and the heterocyclic ring is 74.0 (1).

### Crystal data

**Table:**

C_24_H_21_N_3_O_6_	*D*_x_ = 1.107 Mg m^−^^3^
*M**_r_* = 447.44	Mo *K*α radiation, λ = 0.71073 Å
Trigonal, *R*3*c*	Cell parameters from 1247 reflections
Hall symbol: R 3 -2"c	θ = 2.3–20.4°
*a* = 13.2008 (14) Å	µ = 0.08 mm^−^^1^
*c* = 26.695 (3) Å	*T* = 200 K
*V* = 4028.7 (8) Å^3^	Block, colourless
*Z* = 6	0.51 × 0.49 × 0.04 mm
*F*(000) = 1404	

### Data collection

**Table:**

Bruker SMART CCD area-detector diffractometer	1577 independent reflections
Radiation source: fine-focus sealed tube	1142 reflections with *I* > 2σ(*I*)
Graphite monochromator	*R*_int_ = 0.031
phi and ω scans	θ_max_ = 25.0°, θ_min_ = 2.4°
Absorption correction: multi-scan (*SADABS*; Sheldrick, 1996)	*h* = −9→15
*T*_min_ = 0.960, *T*_max_ = 0.997	*k* = −15→14
6995 measured reflections	*l* = −31→31

### Refinement

**Table:**

Refinement on *F*^2^	Primary atom site location: structure-invariant direct methods
Least-squares matrix: full	Secondary atom site location: difference Fourier map
*R*[*F*^2^ > 2σ(*F*^2^)] = 0.044	Hydrogen site location: inferred from neighbouring sites
*wR*(*F*^2^) = 0.122	H-atom parameters constrained
*S* = 1.05	*w* = 1/[σ^2^(*F*_o_^2^) + (0.0687*P*)^2^] where *P* = (*F*_o_^2^ + 2*F*_c_^2^)/3
1577 reflections	(Δ/σ)_max_ = 0.001
101 parameters	Δρ_max_ = 0.16 e Å^−^^3^
1 restraint	Δρ_min_ = −0.12 e Å^−^^3^

### Special details

**Table:**

Geometry. All e.s.d.'s (except the e.s.d. in the dihedral angle between two l.s. planes) are estimated using the full covariance matrix. The cell e.s.d.'s are taken into account individually in the estimation of e.s.d.'s in distances, angles and torsion angles; correlations between e.s.d.'s in cell parameters are only used when they are defined by crystal symmetry. An approximate (isotropic) treatment of cell e.s.d.'s is used for estimating e.s.d.'s involving l.s. planes.
Refinement. Refinement of *F*^2^ against ALL reflections. The weighted *R*-factor *wR* and goodness of fit *S* are based on *F*^2^, conventional *R*-factors *R* are based on *F*, with *F* set to zero for negative *F*^2^. The threshold expression of *F*^2^ > σ(*F*^2^) is used only for calculating *R*-factors(gt) *etc*. and is not relevant to the choice of reflections for refinement. *R*-factors based on *F*^2^ are statistically about twice as large as those based on *F*, and *R*- factors based on ALL data will be even larger.

### Fractional atomic coordinates and isotropic or equivalent isotropic displacement parameters (Å^2^)

**Table:**

	*x*	*y*	*z*	*U*_iso_*/*U*_eq_	
N1	0.24317 (15)	0.68853 (16)	0.06336 (8)	0.0507 (6)	
O1	0.37628 (17)	0.88360 (16)	0.06161 (9)	0.0710 (6)	
O2	−0.1275 (2)	0.7774 (3)	0.06263 (12)	0.1146 (9)	
C1	0.1470 (2)	0.7108 (2)	0.06541 (11)	0.0552 (7)	
C2	0.0864 (2)	0.6942 (2)	0.10907 (12)	0.0652 (7)	
H2	0.1090	0.6709	0.1387	0.078*	
C3	−0.0104 (3)	0.7124 (3)	0.10944 (13)	0.0768 (9)	
H3	−0.0565	0.6970	0.1388	0.092*	
C4	−0.0369 (2)	0.7525 (3)	0.06691 (14)	0.0724 (8)	
C5	0.0253 (3)	0.7708 (3)	0.02514 (15)	0.0878 (10)	
H5	0.0050	0.7983	−0.0039	0.105*	
C6	0.1168 (3)	0.7514 (3)	0.02308 (12)	0.0729 (8)	
H6	0.1600	0.7654	−0.0071	0.088*	
C7	0.3566 (2)	0.7838 (2)	0.06225 (11)	0.0566 (7)	
C8	−0.2060 (4)	0.7446 (5)	0.1028 (2)	0.1312 (17)	
H8A	−0.2414	0.6604	0.1087	0.197*	
H8B	−0.2674	0.7631	0.0948	0.197*	
H8C	−0.1641	0.7874	0.1329	0.197*	

### Atomic displacement parameters (Å^2^)

**Table:**

	*U*^11^	*U*^22^	*U*^33^	*U*^12^	*U*^13^	*U*^23^
N1	0.0390 (12)	0.0406 (12)	0.0734 (15)	0.0206 (10)	0.0029 (11)	0.0033 (11)
O1	0.0571 (12)	0.0488 (12)	0.1124 (15)	0.0306 (10)	−0.0006 (11)	−0.0036 (10)
O2	0.0943 (19)	0.158 (3)	0.127 (2)	0.0896 (19)	0.0056 (17)	0.0234 (18)
C1	0.0452 (16)	0.0456 (15)	0.0770 (18)	0.0242 (14)	0.0011 (15)	−0.0026 (14)
C2	0.0608 (17)	0.0636 (18)	0.0762 (19)	0.0350 (14)	0.0076 (14)	0.0051 (15)
C3	0.0591 (18)	0.080 (2)	0.089 (2)	0.0331 (16)	0.0207 (15)	0.0075 (17)
C4	0.0538 (16)	0.078 (2)	0.098 (2)	0.0429 (16)	−0.0027 (18)	0.0036 (17)
C5	0.071 (2)	0.111 (3)	0.093 (3)	0.054 (2)	−0.008 (2)	0.0187 (19)
C6	0.0654 (19)	0.079 (2)	0.081 (2)	0.0410 (16)	0.0038 (16)	0.0179 (16)
C7	0.0465 (16)	0.0472 (16)	0.0751 (19)	0.0227 (11)	0.0012 (14)	−0.0021 (14)
C8	0.085 (3)	0.174 (4)	0.166 (4)	0.088 (3)	0.016 (3)	−0.011 (4)

### Geometric parameters (Å, º)

**Table:**

N1—C7^i^	1.384 (3)	C3—C4	1.370 (5)
N1—C7	1.393 (3)	C3—H3	0.9500
N1—C1	1.440 (3)	C4—C5	1.333 (4)
O1—C7	1.209 (3)	C5—C6	1.356 (4)
O2—C4	1.395 (4)	C5—H5	0.9500
O2—C8	1.400 (5)	C6—H6	0.9500
C1—C2	1.368 (4)	C7—N1^ii^	1.384 (3)
C1—C6	1.392 (4)	C8—H8A	0.9800
C2—C3	1.413 (4)	C8—H8B	0.9800
C2—H2	0.9500	C8—H8C	0.9800
			
C7^i^—N1—C7	124.2 (3)	C4—C5—C6	121.7 (3)
C7^i^—N1—C1	117.35 (19)	C4—C5—H5	119.2
C7—N1—C1	118.43 (19)	C6—C5—H5	119.2
C4—O2—C8	117.0 (3)	C5—C6—C1	119.6 (3)
C2—C1—C6	119.7 (2)	C5—C6—H6	120.2
C2—C1—N1	120.3 (3)	C1—C6—H6	120.2
C6—C1—N1	120.0 (2)	O1—C7—N1^ii^	122.2 (2)
C1—C2—C3	119.1 (3)	O1—C7—N1	122.1 (2)
C1—C2—H2	120.5	N1^ii^—C7—N1	115.7 (3)
C3—C2—H2	120.5	O2—C8—H8A	109.5
C4—C3—C2	119.1 (3)	O2—C8—H8B	109.5
C4—C3—H3	120.4	H8A—C8—H8B	109.5
C2—C3—H3	120.4	O2—C8—H8C	109.5
C5—C4—C3	120.7 (3)	H8A—C8—H8C	109.5
C5—C4—O2	114.3 (3)	H8B—C8—H8C	109.5
C3—C4—O2	125.0 (3)		

Symmetry codes: (i) −*y*+1, *x*−*y*+1, *z*; (ii) −*x*+*y*, −*x*+1, *z*.
